# Effects of Family Environment on Depressive Symptoms in Postgraduate Students: Longitudinal Moderating Effect of Family Support and Mediating Effect of Psychological Resilience

**DOI:** 10.1155/da/3867823

**Published:** 2024-12-19

**Authors:** Minxuan Ren, Jingjing Song, Chunyan Zhou, Jinbo Hou, Hai Huang, Lin Li

**Affiliations:** ^1^Department of Psychology, School of Education, China University of Geosciences, Wuhan, China; ^2^Student Mental Health Education Center, China University of Geosciences, Wuhan, China

**Keywords:** depressive symptoms, family environment, family support, longitudinal study, master's and PhD students, psychological resilience

## Abstract

**Background:** Little is known about the mechanism of the relationship between family environment and depressive symptoms in Chinese master's and PhD students. The objective of this study was to investigate the moderating effect of family support and the mediating effect of psychological resilience on depressive symptoms in a family environment. The differences between master's and PhD students are also discussed in this study.

**Methods:** Data for 615 master's and 78 PhD students were collected using the Family Environment Scale, Depression Self-Rating Scale, Perceived Social Support Scale, and Psychological Resilience Scale in October every year for 3 years from 2021 to 2023. A latent growth curve mediation model was used to examine the potential mediating role of psychological resilience in the relationship between family environment and depressive symptoms over time.

**Results:** (1) There was a significant positive correlation among family environment, family support, and psychological resilience and a significant negative correlation between these factors and depressive symptoms. (2) Family support moderated the relationship between family environment and depressive symptoms among both master's and PhD students. (3) Mediation analysis showed that psychological resilience mediated the relationship between family environment and depressive symptoms among master's students.

**Conclusion:** Family environment is a significant risk factor for depressive symptoms. This association appears to be moderated by family support and mediated by psychological resilience. It is necessary to design depressive symptom prevention programs that consider the family environment of master's and PhD students. Therefore, mental health services for this population should consider both family support and psychological resilience.

## 1. Introduction

In recent years, news about the emergence of mental health problems among master's and PhD students has emerged in China. Research has shown that the mental health status of master's students is far poorer than that of Chinese adults [1]. This group has a high incidence of mental health problems [2], which may be as minor as showing signs of depression and anxiety [3] or ending their studies for mental health reasons [4] or as serious as endangering their lives [5, 6]. Moreover, one survey found that of 1263 PhD students in the United Kingdom, 40% showed a high risk of suicide [7], and 48.73% of Chinese PhD students showed mild-to-severe levels of depressive symptoms [8]. This recent series of issues has sparked widespread social concern regarding the psychological well-being of master's and PhD students. In addition to individual factors, in terms of environmental influences on master's and PhD students' mental health, previous studies have analyzed the effects of school, family, interpersonal environment [9–11], supervisors [12], research climate [13], and sociocultural context [14] on mental health.

Depression is an important risk factor for mental health and can greatly harm quality of life and social adjustment of adults [15] and Chinese university freshman [16]. In a sample of adult students, previous studies found that over one-fourth of Chinese university undergraduate students experienced depressive symptoms [5, 6], and 39% of Chinese postgraduate students represent mild, moderate, and severe depressive symptoms [17] during the COVID-19 pandemic. Moreover, the incidence of depression among master's and PhD students is six times higher than that among the general population [12]. Additionally, a report on China's national mental health development highlighted that 35.5% of master's students exhibit symptoms of depression. Depressive symptoms show an upward trend from the master's to the PhD stage in China [18], which not only impacts students' quality of life but also hinders their social cognitive functions [19] and can even lead to suicidal risk or behavior [20].

### 1.1. Family Environment and Depressive Symptoms

Exploration and research on the mechanism of depressive symptoms have been ongoing for many years in the academic world, and family environment is an important influencing factor [21]. Family is the basic social unit in which individuals live, and the family atmosphere affects the mental health of family members [22]. Previous studies have shown that the family environment can directly affect family members' emotions [23]. Ecological systems theory (EST) states that in the microsystem, the family has the greatest influence on the individual [24] and that this influence lasts for a long time because the family is an important living environment for the individual in the process of growing up. The family environment directly or indirectly affects an individual's psychological condition and behavior. Based on Erickson's eight-stage theory, most master's and PhD students are in early adulthood. The transition from family dependence to independence and socialization is extremely challenging. In addition, because of the popular authoritative parenting style and the necessity of family connection in the Chinese cultural context, master's students in early adulthood are still affected by the family environment [25]. Although they have just been separated from their families and have gained a certain degree of freedom, the family environment that once accompanied their growth will continue to affect the psychological health of master's students [26]. Family conflict is a significant source of mental health risk for master's and PhD students [13]; individuals who experience poor family relationships report more depressive symptoms [27], and experiencing a difficult family environment induces stronger depression [28].

### 1.2. The Moderating Role of Family Support

The family environment is a complex and dynamic construct that includes a variety of domains, such as socioeconomic status, household composition and functioning, parenting behaviors, mental health, and interactional patterns [29]. The influential role of the family environment is generally divided into two aspects: a harder, adverse, and unfavorable family environment (i.e., family closeness, family income, and parents' occupation) leads to individuals being more aggressive [9, 11], triggering self-injurious behaviors [30], generating depression, and possibly inducing nonsuicidal self-injurious behaviors [31]. On one hand, family members tend to reject their children's positive emotions [32]; on the other hand, they are also less able to accept their children's negative emotions [33] when they have difficulty regulating those emotions, which may gradually evolve into depression. A better, softer, and more favorable family environment (i.e., a harmonious family atmosphere and democratic education) can lead individuals to generate more positive psychological capital [34], recover faster from trauma [35], and obtain and perceive more abundant social support [36].

Although an unfavorable family environment may create some disadvantages in an individual's development and mental health status, it is not necessarily the case that individuals in difficult family environments will have high levels of depressive symptoms, and some protective factors can help individuals in such family environments develop in a healthy manner. These factors can come from both external sources (e.g., family support) and internal sources (e.g., psychological resilience). The social support buffer model posits that social support acts as a protective factor, often through a person's internal cognitive system, which exerts a positive effect on psychological traits [37] and acts as a buffer between the family environment and depression [38]. Perceived family support is an important dimension of perceived social support, which refers to perceived help and support from family members [39]. Not only is it closely related to the family environment, but the amount of perceived family support an individual will continue to influence mental health status [40]. Individuals with high levels of family support have more external support resources and can adjust their emotions and adapt to the situation faster; thus, it is an important external protective factor for the mental health of primary medical staff [41]. Moreover, family support directly moderates the relationship between interpersonal sensitivity and depression when it is sensed and utilized by individuals [42]. Individuals with low family support are unable to adjust their emotions quickly or at all, resulting in lower self-esteem and more depressive symptoms [43, 44]. A meta-analysis also showed that family support was significantly negatively correlated with depression among college students in China [45].

### 1.3. The Mediating Role of Psychological Resilience

Psychological resilience, as an ability and quality, refers to an individual's ability to recover quickly in the face of stress and adversity and to adapt flexibly to the changing external environment [46] and is inextricably linked to an individual's mental health status. A machine learning approach showed that mental health and psychological resilience were related during the coronavirus pandemic [47] and that there was a significant positive correlation between mental resilience and the mental health of medical master's degree students [48]. Previous studies have shown that psychological resilience was negatively correlated with depression among Indian undergraduate students [49]. It happens that there are some similar cases and resilience was also negatively correlated with depressive symptoms among the general population in China [50] and Chinese undergraduate students [51] during the pandemic. This correlation may be bidirectional: on one hand, the level of psychological resilience affects the ability of individuals to quickly regulate their depressive symptoms; on the other hand, depressive symptoms have a negative impact on the level of psychological resilience, which may aggravate the level of depressive symptoms. At the same time, there is a positive correlation between family environment and psychological resilience [52].

Therefore, psychological resilience may be a key protective factor in the mechanism of the family environment's effect on the level of depressive symptoms in master's and PhD students. Family environment profoundly affects the level of individual mental resilience, which has an important effect on depressive symptoms in these students. Based on this, one of the focuses of the present study is to investigate whether psychological resilience exists as a mediating mechanism between family environment and depressive symptoms in master's and PhD student groups and whether there is a bidirectional relationship between psychological resilience and depressive symptoms in master's and PhD students in more stable family environments. This shift necessitates a deeper understanding of the connection among family environment, psychological resilience, and depressive symptoms from a developmental perspective. Latent growth curve modeling describes the trajectory of change at several points in time, tracing developmental trajectories by considering initial levels and changes and expecting relatively large intraindividual changes. Therefore, it is particularly suitable for mediating [53] and growth curve mediating analyses with time-invariant independent variables [54]. Therefore, this study employed a longitudinal design and latent growth curve modeling to investigate the direct and indirect relationships between the initial level of family environment (stable and not changing over time) and developmental trajectories of psychological resilience and depressive symptoms.

### 1.4. The Present Study

As the master's and PhD populations grow in size, mental health is receiving increasing attention. Adopting practical approaches to improve the mental health of master's and PhD students can help cultivate high-quality and highly educated groups. Most previous studies have examined these relationships from a horizontal perspective, making it difficult to examine developmental changes, bidirectional relationships, causal relationships between phenomena and behaviors, and differences between master's and PhD students. From a bioecological perspective, an individual's behavior is influenced by multiple ecological subsystems [55]. Analogously, developmental psychopathology posits that an individual's internal resources, external environment, and interactions affect the developmental process of an individual's mental illness [56]. Therefore, attention should be paid to the synergistic effects of factors in different domains on individual master's and PhD students' family environments, perceived family support, psychological resilience, and depressive symptom levels, which fluctuate at different time stages. This study used a longitudinal design with master's and PhD students and examined the potential moderating effect of family support using a latent growth curve analysis to test the relationship between the initial levels of the family environment and changes in depressive symptoms and the potential mediating role of the change in psychological resilience. Based on the theory and studies reviewed above, we propose the following hypotheses:

**Hypothesis 1 .**

Family environment has a significant negative predictive effect on current and later depressive symptoms in master's and PhD students.

**Hypothesis 2 .**

Perceived family support can effectively cushion the negative impact of the family environment on depressive symptoms in master's and PhD students, and this moderating effect exists over time.

**Hypothesis 3 .**

Psychological resilience mediates the association between the family environment and depressive symptoms of master's and PhD students, and this mediating role exists across time.

This study was aimed at first-year master's and PhD students and collected data once a year for 3 years to explore the influencing factors and longitudinal mechanisms of the family environment on depressive symptoms of these students.

## 2. Materials and Methods

### 2.1. Samples and Procedures

Data for this analysis were derived from a questionnaire distributed among Chinese master's and PhD students in a three-wave prospective study conducted from 2021 to 2023 in Hubei Province, China. Using the cluster sampling method, we administered the test to master's and PhD students of a university in Wuhan City, Hubei Province, in October 2021, October 2022, and October 2023, with an interval of 1 year between administrations. It should be noted that to have a clearer understanding of the mood changes of the study participants at the time of enrollment and graduation, only first-year students who had just enrolled in the master's and PhD programs at that time were selected for the first measurement in October 2021. By the time of the measurement in 2023, they had already reached their graduation year.

At baseline (T_1_), after removing invalid questionnaires, 1672 first-year master's and PhD students (47.01% female, M_age_ = 22.86, SD = 2.06) were followed up with for subsequent investigation. Specifically, there were 1452 master's students (47.93% female, M_age_ = 22.39, SD = 1.39) and 220 PhD students (40.91% female, *M*_age_ = 25.96, SD = 2.90). At T_2_ (1 year after baseline) and T_3_ (2 years after baseline), after excluding invalid data (not answering all questions completely), the percentages of participants remained at 73.15% and 41.48%, respectively. The main reason for attrition was that the second and third measurements were in the form of an online questionnaire (the first was offline, and the test was completed using the provided computer), so it is possible that they did not see the notification or forgot about it, among other reasons. Specifically, 1087 master's students (49.22% female, M_age_ = 23.57, SD = 1.35) and 136 PhD students (42.65% female, M_age_ = 26.96, SD = 2.92) participated in the second online survey. However, 615 master's students (53.66% female, M_age_ = 24.58, SD = 1.45) and 78 PhD students (44.87% female, M_age_ = 27.94, SD = 2.96) were ultimately retained as valid subjects. There were 615 (88.74%) master's degree students and 78 (11.26%) PhD students among the effective subjects: 365 (52.67%) were male and 328 (47.33%) were female.

This study was approved by the Ethics Committee, Department of Psychology, China University of Geosciences (cug-ecdp-21-09-01), and we also emphasized to the students that their participation was voluntary and they could withdraw from the study at any time without repercussions.

### 2.2. Measures

#### 2.2.1. Family Environment

The Family Environment Scale was compiled by Moos et al. [57] and translated and revised by Fei et al. [58]. The scale consists of 90 items across 10 dimensions: intimacy, emotional expression, ambivalence, independence, success, knowledge, recreation, moral and religious views, organization, and control. All items were scored according to the answers chosen, with “yes” scoring one point and “no” scoring two points. The total score for family environment was the sum of the scores of the 10 dimensions (the scores of the ambivalence subscale were reverse-transformed); the higher the total score of the family environment was, the better the family environment. The Cronbach's alpha value for this scale in this study was 0.735, indicating good reliability [59].

#### 2.2.2. Depressive Symptoms

The Self-Rating Depression Scale was developed by Zung [60]. The 20-item scale examines how individuals are feeling right now or felt during the past week. The scale is rated on a four-point Likert scale ranging from 1 to 4, representing “no or very little time” to “most or all of the time.” The scale consisted of 10 positively scored items and 10 negatively scored items. The scores of all items were added together to obtain a total crude score X, which was then converted using the formula *Y* = in + (1.25*X*). That is, multiplying the crude score by 1.25 and then taking the integer part yield the standardized score *Y*. Higher scores indicate more severe depressive symptoms. In the current study, Cronbach's alpha values measured three times were 0.852, 0.860, and 0.869, respectively, indicating good internal consistency [60].

#### 2.2.3. Family Support

The Chinese version of the Zimet Perceived Social Support Scale (PSSS) was developed by Blumenthal in 1987 and later translated and modified by Jiang [61]. The scale was used to assess the degree of perceived support from family, friends, and significant others, with four questions in each dimension and 12 questions in total. A seven-point scale was used, with higher scores representing higher levels of perceived support. In the current study, the family support dimension of the Perceived Support Scale was selected to test the participants, including four items such as “My family can help me in a concrete way.” According to previous research [61], the Cronbach's alpha values for family support in the current study were 0.891, 0.895, and 0.893, respectively, indicating good confidence.

#### 2.2.4. Psychological Resilience

The Chinese version of the Connor–Davidson Resilience Scale (CD-RISC), which was translated and revised by Yu and Zhang, was used for the assessment [62]. The scale includes three dimensions—optimism, resilience, and strength—with 25 items. A five-point Likert scale was used, with scores ranging from 0, “not at all,” to 4, “almost always,” and the total score was 100, with higher scores reflecting more psychological resilience. At T_1_ of this study, the Cronbach's alpha value of the questionnaire in general and for each dimension was 0.941, while it was 0.952 for T_2_ and 0.965 for T_3_. According to previous studies [62], the coefficients of the three time points were good.

#### 2.2.5. Covariates

Gender at T_1_ was controlled for as a time-invariant covariate in all models, as this variable has been found to be related with depressive symptoms among Chinese master's and PhD students [63].

### 2.3. Statistical Analysis

The preliminary analysis was divided into six steps. First, we performed a common method deviation analysis, descriptive statistical analysis, correlation analysis, and one-way ANOVA using SPSS (version 22.0 for Windows, Armonk, NY: IBM Corp.), with statistical significance set at *p* < 0.05 (two-sided). Second, we then examined the reliability of the four scales at three time points. Third, Process version 3.4 [64] compiled by Hayes in SPSS was used to test the moderating role of family support. Fourth, the unconditional potential growth model was constructed using Mplus version 8.0 to examine the developmental trend of mediating and dependent variables separately, with the intercept and slope representing the starting state and development rate, respectively. Fifth, a series of univariate latent growth curve models (LGCMs) were constructed using three sets of psychological resilience data to examine whether the developmental trajectory of depressive symptoms was directly affected by the family environment. Finally, the relationships between the intercepts and slopes of family environment, psychological resilience, and depressive symptoms were explored using structural equation modeling, and the significance of the mediating effects was further verified using the bootstrap method. Models were considered acceptable when the chi-square and degree of freedom ratio (*χ*^2^/df) was below 8, the comparative fit index (CFI) and Tucker–Lewis Index (TLI) were above 0.90, and the standardized root mean square (SRMR) and root mean square error of approximation (RMSEA) were below 0.10 [65].

## 3. Results

### 3.1. Attrition Analyses

According to a previous study [66], chi-square and *t*-tests were used to compare demographic characteristics and outcome variables at baseline between participants who completed the three surveys and those who did not. There were no significant differences between attrition subjects and valid subjects for family environment (*t* = −0.10, *p* > 0.05), level of depressive symptoms (*t* = −0.84, *p* > 0.05), perceived family support (*t* = 0.45, *p* > 0.05), or psychological resilience (*t* = −0.29, *p* > 0.05) at T_1_, indicating that the subjects reflected unstructured attrition. Moreover, the results showed that male participants were more likely to drop out than female participants (*χ*^2^ = 14.45, *df* = 1, *p* < 0.001).

### 3.2. Common Method Bias Analysis

As this study used a questionnaire method, there may be a problem of common method bias; therefore, a statistically controlled method was adopted to conduct the common method bias test. All of the question items were statistically tested using the Harman single-factor analysis; that is, if only a single factor was analyzed or if a certain factor had particularly high explanatory power, then it was judged that there was serious common method bias. After importing all data of the three measurements into SPSS 22, the single-factor analysis test method was used to integrate all measurement topics into one variable, and the principal component analysis method was used to extract 55 factors with characteristic roots >1. The first principal component factor explained 16.77% of the variation, which was <30% of the variation interpretation [67]. The data showed that the common method deviation did not have a significant impact on the conclusions.

### 3.3. Descriptive Analyses and Correlation Analysis

Descriptive statistical analyses and correlation matrices for each research variable are presented in Table 1. Depressive symptoms were negatively correlated with family environment, family support, and psychological resilience at all time points. Family environment was positively correlated with social support and psychological resilience at three time points.

### 3.4. A Test of the Moderating Role of Family Support

The moderating role of family support in the relationship between family environment and depressive symptoms was tested first. Considering the differences between grades, we distinguished between master's and PhD students. The 3-year total scores for family support and depressive symptoms were then added together and divided by three to get the 3-year average. The moderating effect was examined using calculated means of family support and depressive symptoms.

**Figure 1:**
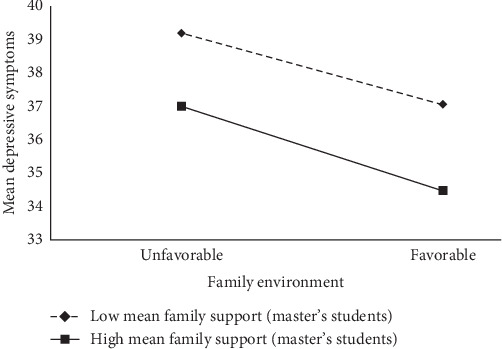
Simple slopes for family environment and mean depressive symptom relations at different mean family support levels among master's students.

The results after controlling for variables of gender (Table 2) showed that the main effects of family environment (*β* = −0.11, *p* < 0.01) and mean family support (*β* = −1.22, *p* < 0.001) were significant, and both of them negatively predicted the level of depressive symptoms for master's students; the negative prediction of depressive symptoms by family environment × mean family support was significant (*β* = −0.02, *p* < 0.01), suggesting that for master's students, family support plays a moderating role in family environment and depressive symptoms. Further simple slope analyses showed (Figure 1) that the negative prediction of depressive symptoms by family environment was not significant at low mean family support (simple slope = −0.04, *t* = −0.96, *p* > 0.05) and that the negative prediction of depressive symptoms by family environment was strengthened at high mean family support (simple slope = −0.18, *t* = −3.68, *p* < 0.001). Individuals with low family support were more likely to be depressed and had higher levels of depressive symptoms than those with high family support for the same level of family environment. There was a more significant reduction in the levels of depressive symptoms in the high family environment situation than in the low family environment situation, and the effect was more pronounced for high family support, suggesting that family support plays an enhanced role in the negative prediction of depressive symptoms by family environment.

**Figure 2:**
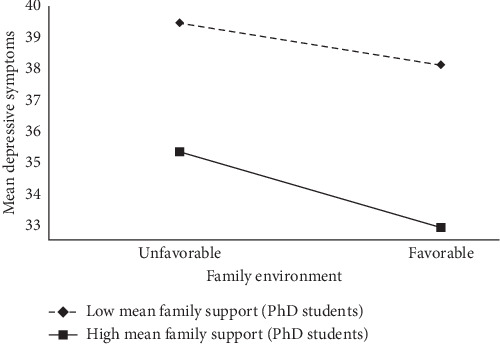
Simple slopes for family environment and mean depressive symptom relations at different mean family support levels among PhD students.

To verify the cross-temporal stability of family support in the relationship between family environment and depressive symptoms in PhD students, whether family support moderates the relationship between family environment and depressive symptoms was examined. Results controlling for gender (Table 3) showed nonsignificant main effects of family environment (*β* = −0.07, *p* > 0.05) and significant mean family support (*β* = −1.19, *p* < 0.001), both of which negatively predicted the level of depressive symptoms and a significant negative predictive effect of family environment × mean family support on depressive symptoms (*β* = −0.05, *p* < 0.05), indicating that family support plays a moderating role in family environment and depressive symptoms. Simple slope analyses indicated (Figure 2) that for PhD students with low mean family support, the negative prediction of depressive symptoms by family environment was not significant (simple slope = 0.12, *t* = 0.89, *p* > 0.05), whereas for PhD students with high family support, the negative prediction of depressive symptoms by family environment was stronger (simple slope = −0.26, *t* = −1.99, *p*=0.051), suggesting that the negative predictive effect of the family environment on the depressive symptoms of individual PhD students showed an upward trend as their appreciation of family support increased.

### 3.5. A Test of the Mediating Role of Psychological Resilience

#### 3.5.1. The Trajectory of the Mediating and Dependent Variables

All of the univariate LGCMs provided a good fit for the data. Detailed information is presented in Table 3. For master's students, the mean intercepts for psychological resilience and depressive symptoms were significant, representing both psychological resilience and depressive symptoms at the baseline level, and were significantly greater than 0. The mean slopes for psychological resilience and depressive symptoms were significant and positive, indicating that psychological resilience and depressive symptoms tended to increase linearly across the three follow-up measures over 3 years. Among PhD students, the mean values of the intercepts for psychological resilience and depressive symptoms were significant. However, none of the slopes were significant, indicating that the trend of psychological resilience and depressive symptom levels of PhD students did not reach significance over the 3 years.

#### 3.5.2. The Direct Effect of Family Environment on the Onset Level and Rate of Development of Depressive Symptoms

**Figure 3:**
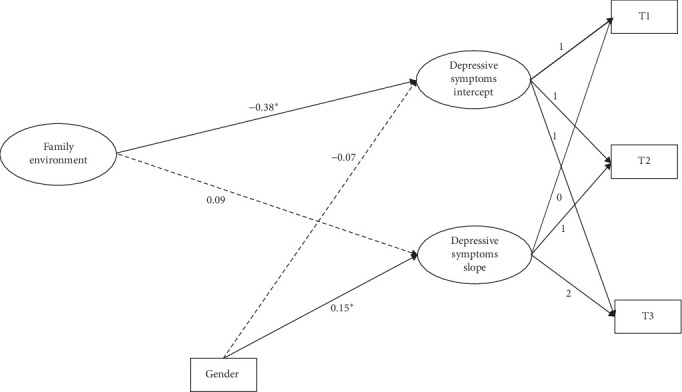
The influence of family environment on the trajectory of depressive symptoms in master's students. *⁣*^*∗*^*p* < 0.05.

**Figure 4:**
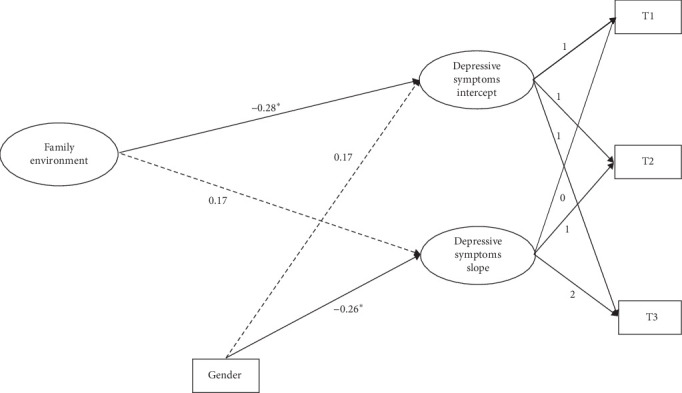
The influence of family environment on the trajectory of depressive symptoms in PhD students. *⁣*^*∗*^*p* < 0.05.

By controlling for gender, a conditional linear growth model was constructed to test the effect of family environment on the development of depressive symptoms. For the results for master's students (Figure 3), the model fitted well (*χ*^2^/df = 5.92, CFI = 0.96, TLI = 0.88, SRMR = 0.05, RMSEA = 0.09). For PhD students (Figure 4), the model also fitted well (*χ*^2^/df = 1.44, CFI = 0.99, TLI = 0.95, SRMR = 0.03, RMSEA = 0.08). As can be seen from the results, for both master's and PhD students, the family environment significantly and negatively predicted the intercept of depressive symptoms but had no significant effect on the slope of depressive symptoms. This indicates that individuals with better baseline family environments have lower levels of depressive symptoms. The increase in depressive symptom levels was not related to the family environment of individuals with master's degrees or PhD students.

#### 3.5.3. Latent Growth Curve Mediation Models

**Figure 5:**
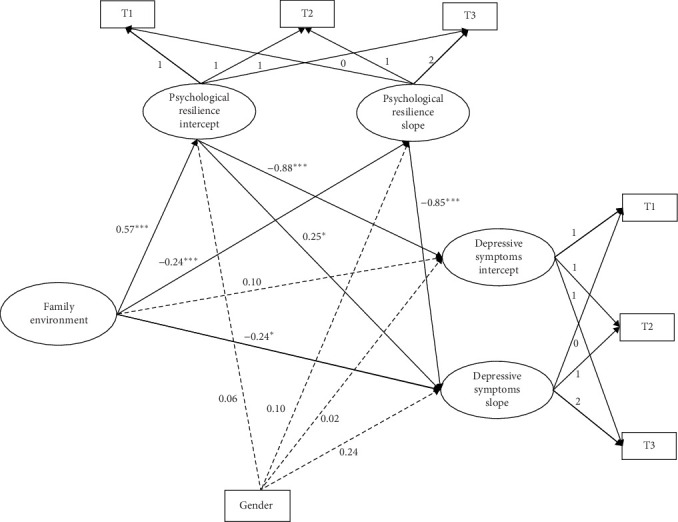
Parallel process latent growth curve mediation model for family environment, psychological resilience, and depressive symptoms in master's students. *⁣*^*∗*^*p* < 0.05, *⁣*^*∗∗∗*^*p* < 0.001.

Based on the hypotheses and analysis of the relationships between the variables, a latent growth curve mediation model was developed for each group to test the mediating role of psychological resilience. It was found that the model fits for both master's (*χ*^2^/df = 10.89, CFI = 0.91, TLI = 0.82, SRMR = 0.05, RMSEA = 0.13) and PhD students (*χ*^2^/df = 1.60, CFI = 0.96, TLI = 0.92, SRMR = 0.11, RMSEA = 0.09) were almost within acceptable limits. The direct paths between family environment, psychological resilience, and depressive symptoms, controlling for gender, are shown in Table 4. For master's students, the initial level of the family environment was significantly positively associated with the intercept of psychological resilience, which, in turn, was associated with the intercept of depressive symptoms. Similarly, family environment was positively associated with the rate of change (slope) of psychological resilience, which, in turn, was associated with the slope of depressive symptoms (Figure 5).

Master's students with higher initial levels of psychological resilience had lower initial levels of depressive symptoms and a faster downward trend in depressive symptoms at follow-up. Based on the good simulation fit and significant direct paths of the longitudinal mediation model, the mediating role of psychological resilience intercept and slope was further verified using the bootstrap method with 2000 repeated samples. The standardized estimates and 95% confidence intervals for the mediating effects of each indirect path are presented in Table 5. The results showed that all three indirect paths were significant, indicating that the family environment of master's students can indirectly influence the level of depressive symptoms and that the developmental trajectory of psychological resilience played a fully longitudinal mediating role in the association between the developmental trajectories of the family environment and depressive symptoms.

**Figure 6:**
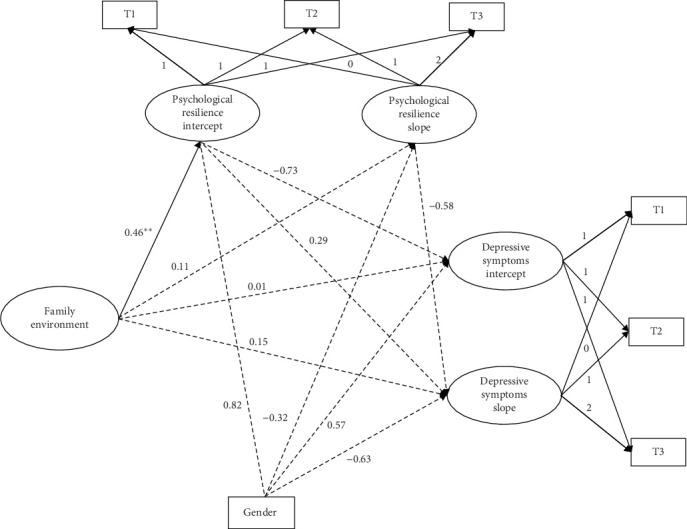
Parallel process latent growth curve mediation model for family environment, psychological resilience, and depressive symptoms in PhD students. *⁣*^*∗∗*^*p* < 0.01.

For PhD students (Figure 6), the direct effects of the family environment, psychological resilience, and depressive symptoms were not significantly correlated. Similarly, none of the three indirect paths were significant, suggesting that psychological resilience could not play a longitudinal mediating role between the family environment and depressive symptoms in PhD students.

## 4. Discussion

The family is an important place and environment for individual development, and the quality of the family environment is related to the psychological health of individuals [68]. If individuals are exposed to a poor family environment for a long time while growing up and their needs for security and intimacy are not met, it may lay the foundation for their poor emotional state. When exploring the relationship between the family environment and depressive symptoms, previous studies have often focused on adolescents or college students with a larger base. The current study used master's and PhD students as research subjects and systematically examined the relationship between their family environment and depressive symptoms by using longitudinal tracking at 1-year intervals. Family environment was found to have a significant negative predictive effect on subsequent depressive symptoms, which supports H1. This study also found that family support played an immediate and longitudinal moderating role in the relationship between the family environment and depressive symptoms among master's and PhD students. Thus, it provides important insight into the key psychological mechanism through which psychological resilience plays an immediate and longitudinal mediating role between master's students' family environment and depressive symptoms.

### 4.1. The Potential Moderating Role of Family Support Between Family Environment and Depressive Symptoms

This study found that, from a longitudinal perspective, perceived family support plays a moderating role between master's and PhD students' family environments and depressive symptoms; that is, perceived family support can effectively buffer the negative impacts of the family environment on individual master's and PhD students' depressive symptoms, which validates H2. This suggests that, although a poor family environment negatively affects master's and PhD students' level of psychological well-being, leading to depressive symptoms, if individual students can receive and perceive the support given to them by their family members, it will buffer the negative effects of depressive symptoms, especially for individual students who have been growing up in a good family environment. Their perceived family support is generally higher, which adds to the effect of depressive symptoms and significantly reduces the level of depressive symptoms.

Longitudinal studies have shown that, for master's students, low levels of family support do not influence the impact of the family environment on depressive symptoms. In contrast, for PhD students, the perceived level of family support does not enhance the buffering effect of the family environment on depressive symptoms. The reasons for this may lie, first, in the perspective of the stress vulnerability model. In high-stress environments, the positive factors lose their buffering effects. Family support, to some extent, reflects an individual's family situation, and lower family support may be accompanied by higher family stress; therefore, master's students who understand that the resilience of family support, which is supposed to be a positive factor, decreases rapidly or even disappears when they can only perceive less or even no family support [69] and is unable to withstand the negative effects of the family environment on depressive symptoms. Second, according to Beck's cognitive theory, postgraduate students who shows depressive symptoms may have cognitive biases that cause them to tend to ignore positive information and pay more attention to negative information [70]. As a result, they are less likely to actively receive family support, which prevents family support from moderating the relationship between family environment and depressive symptoms. Third, PhD students are older, and the influence of the family environment on their individuality weakens in preadulthood. This may be because most PhD students are already married and have families of their own and what they perceive as family support may not be equivalent to a growing family. At the same time, PhD students face greater pressure from academic and research stress, interpersonal relationships, and future development. Under these circumstances, even in better family environments, they do not show lower levels of depressive symptoms.

### 4.2. The Potential Mediating Role of Psychological Resilience in the Relationship Between Family Environment and Depressive Symptoms

The present study found an overall linearly increasing trend in psychological resilience and the development of depressive symptoms in master's degree students over a 3-year follow-up. The onset level of family environment directly predicted the onset and rate of depressive symptoms to varying degrees. The development of psychological resilience can also have an indirect effect on the development of depressive symptoms in master's degree students through the onset level of the family environment. This result validates H3 and confirms the integrative model of psychological resilience [71], which suggests that the addition of protective factors to the development of psychological resilience can be effective in facilitating individuals' positive modification and adaptation to negative circumstances. For PhD students, the trends of psychological resilience and depressive symptoms were nonlinear, and psychological resilience was unable to play an indirect role in the family environment and depressive symptoms. This may be because, first, the number of PhD students was much smaller than that of master's students, leading to results that may have been somewhat affected. Second, this study found a downward trend in psychological resilience among PhD students. This is similar to the depression model of cognitive vulnerability, which suggests that if an individual holds a negative view of the self, they will adopt a negative attitude toward the future and that poorer family environments lead more directly to depression [72].

Notably, in the direct pathway, the family environment of individuals with master's degrees negatively predicted psychological resilience. This may be because the development of psychological resilience may be curvilinear in relation to being more influenced by factors outside the family in adulthood and high psychological resilience due to the family environment may have shown a weak regression to the mean. Second, psychological resilience in early adulthood is not only influenced by the family environment. At the baseline level, the family environment and psychological resilience change in the same direction; however, as individuals grow older, they are also affected by other factors such as intimate relationships, academic pressures, and interpersonal relationships during graduate school, which leads to an increasing trend of psychological resilience and a negative change in the starting level of the family environment.

The main aim of this study was to reveal the longitudinal mediating role and structural dynamic properties of psychological resilience between family environment and depressive symptom development. Based on the significance of the direct path, we tested the indirect path in a longitudinal mediation model using the bootstrap method. All three indirect paths were valid in the postgraduate group, where psychological resilience played a longitudinal mediating role in the influence of the family environment on the mechanisms of depressive symptom development. On one hand, according to the ecosystem theory, the family environment is an important microsystem for the physical and mental development of an individual, and its specific situation has a profound and long-lasting influence on the individual. Master's degree students who grow up in good family environments usually have higher psychological resilience and are able to utilize their inner resources to face stressful situations, whereas master's degree students who grow up in poorer family environments tend to have weaker inner resources and are more likely to develop negative emotions when facing adversity in early adulthood, which can lead to an increase in depressive symptoms. In the “richer is richer, weaker is weaker” model [73], master's students who grew up in good family environments had higher levels of psychological resilience and higher levels of protection of self-resources and emotional states, whereas master's students who grew up in poorer family environments had lower levels of psychological resilience and protection of the self. On the other hand, individuals with high psychological resilience experience better physiological and negative emotional recovery from stressful situations [74], are more skilled and accustomed to using positive emotions [75] for stress adaptation, and can attenuate the effects of poor family environments on levels of depressive symptoms. Low psychological resilience tends to be accompanied by high levels of negative emotions, which increase the levels of depressive symptoms. Even though the effects of the family environment on individual graduate students in the early stages of adulthood are long lasting, the ability to tap into or cultivate positive psychological qualities, such as psychological resilience, from the perspective of the individual graduate student can help individual graduate students increase positive perceptions of the self, improve the level of positive emotions and internal resources of the individual, and shorten the period of psychological stress, which can further improve the individual graduate students' ability to cope with adverse family environments and weaken the adverse effects of the family environment on depressive symptoms.

### 4.3. Strengths and Limitations

This study has certain strengths, including special objects, a large sample size, a long-term follow-up survey, and a novel statistical model. The latent growth model was used to accurately describe both the initial levels and developmental trajectory, characteristics, and dynamic mechanisms of depressive symptoms and risk factors in master's and PhD students. This approach enabled a better exploration of the causal and vertical links between the variables. Moreover, considering that the study was conducted during the COVID-19 pandemic, it suggests that the COVID-19 pandemic may have indirectly influenced the high level of mental healthcare of Chinese master's and PhD students in the present and future.

Despite these strengths, several limitations should be considered when interpreting the results. First, the whole population sampling in this study had a large sampling error, and it is necessary to expand the scope of the sampling survey to improve the representativeness and applicability of the conclusions in related follow-up research. Second, the short tracking period and low number of rounds made it difficult to systematically analyze the long-term trends of the variables. In fact, many master's and PhD students do not graduate in their third year. Therefore, future research should conduct multiple instances of tracking over a long period of time to obtain a more accurate long-term trajectory of the variables. Third, this study only used on-site measurements in the first year of administration. In the future, the reliability of the findings can be strengthened using an offline form of data collection in each case. Fourth, the large difference in the number of master's and PhD students in this study may have led to data errors. Fifth, all of the variables were collected based on self-report. Finally, this study explored only the unidirectional predictive roles of these variables. Future research could add cross-lagged models to mine the bidirectional relationships.

## 5. Conclusion

The present study indicated that the influence of master's and PhD students' family environments on their individual depressive symptoms is persistent. Additionally, for both master's and PhD students, family support can play a sustained protective role against the influence of the family environment on individual depressive symptoms during the academic period. Finally, the initial level and development rate of psychological resilience play a longitudinal mediating role in the influence of the family environment on the development of depressive symptoms. Given that depressive symptoms result from the family environment, family members should create a favorable and safe family atmosphere as much as possible. Family members need to express their emotions more appropriately in the course of getting along with each other, avoiding and reducing conflicts and overcontrol, and providing more family support. At the same time, schools should also pay attention to students' family situations and teach them the atmosphere and methods of coping with adverse family environments. For master's and PhD students with depressive symptoms, breathing anchor mediation and body scans can be used to reduce depressive symptoms, and mindfulness-based stress reduction (MBSR) to improve mental health and quality of life [76]. On the other hand, because psychological resilience is considered an important mechanism in the relationship between the home environment and depressive symptoms, psychological resilience processing training may be beneficial. Psychological resilience training in the five areas of cognitive, mental, behavioral, social competence, and physical helps individuals recognize the deficits that exist so that they can better cope with depression, enhance self-regulation, and improve mental health [48].

## Figures and Tables

**Table 1:** Descriptive statistics and bivariate correlations among primary study variables.

Variables	1	2	3	4	5	6	7	8	9	10	11
1. Gender	1	—	—	—	—	—	—	—	—	—	—
2. FE (T_1_)	0.03	1	—	—	—	—	—	—	—	—	—
3. DS (T_1_)	−0.05	−0.33*⁣*^*∗∗*^	1	—	—	—	—	—	—	—	—
4. FS (T_1_)	−0.03	0.46*⁣*^*∗∗*^	−0.48*⁣*^*∗∗*^	1	—	—	—	—	—	—	—
5. PR (T_1_)	0.11*⁣*^*∗∗*^	0.49*⁣*^*∗∗*^	−0.56*⁣*^*∗∗*^	0.51*⁣*^*∗∗*^	1	—	—	—	—	—	—
6. DS (T_2_)	−0.01	−0.22*⁣*^*∗∗*^	0.54*⁣*^*∗∗*^	−0.28*⁣*^*∗∗*^	−0.41*⁣*^*∗∗*^	1	—	—	—	—	—
7. FS (T_2_)	−0.03	0.37*⁣*^*∗∗*^	−0.35*⁣*^*∗∗*^	0.57*⁣*^*∗∗*^	0.34*⁣*^*∗∗*^	−0.47*⁣*^*∗∗*^	1	—	—	—	—
8. PR (T_2_)	0.05	0.33*⁣*^*∗∗*^	−0.44*⁣*^*∗∗*^	0.32*⁣*^*∗∗*^	0.57*⁣*^*∗∗*^	−0.58*⁣*^*∗∗*^	0.52*⁣*^*∗∗*^	1	—	—	—
9. DS (T_3_)	0.03	−0.23*⁣*^*∗∗*^	0.39*⁣*^*∗∗*^	−0.22*⁣*^*∗∗*^	−0.29*⁣*^*∗∗*^	0.51*⁣*^*∗∗*^	−0.31*⁣*^*∗∗*^	−0.40*⁣*^*∗∗*^	1	—	—
10. FS (T_3_)	−0.09*⁣*^*∗*^	0.30*⁣*^*∗∗*^	−0.32*⁣*^*∗∗*^	−0.43*⁣*^*∗∗*^	0.27*⁣*^*∗∗*^	−0.31*⁣*^*∗∗*^	0.53*⁣*^*∗∗*^	0.40*⁣*^*∗∗*^	−0.43*⁣*^*∗∗*^	1	—
11. PR (T_3_)	−0.01	0.25*⁣*^*∗∗*^	−0.28*⁣*^*∗∗*^	−0.23*⁣*^*∗∗*^	0.42*⁣*^*∗∗*^	−0.33*⁣*^*∗∗*^	0.34*⁣*^*∗∗*^	0.56*⁣*^*∗∗*^	−0.46*⁣*^*∗∗*^	0.55*⁣*^*∗∗*^	1
M	0.47	56.75	42.38	22.86	67.37	44.21	21.74	66.33	43.44	22.2	71.3
SD	0.5	9.88	9.75	3.91	13.52	10.68	4.44	15.92	10.85	4.3	16.61

*Note:* Gender is a dummy variable; boy = 1; girl = 0.

Abbreviations: DS, depressive symptoms; FE, family environment; FS, family support; M, mean; PR, psychological resilience; SD, standard deviation.

*⁣* ^*∗∗*^ *p* < 0.01, *⁣*^*∗*^*p* < 0.05.

**Table 2:** The moderating role of family support on the relationship between family environment and depressive symptoms.

Regression equation		Fitting index			Coefficient significance	
Dependent variable	Independent variable	*R*	*R^2^*	*F*	*β*	*t*
Master's students (*n* = 615)						
Mean DS	Gender	0.55	0.30	65.65*⁣*^*∗∗∗*^	−0.64	−1.13
	FE	—	—	—	−0.11	−3.17*⁣*^*∗∗*^
	Mean FS	—	—	—	−1.22	−12.96*⁣*^*∗∗∗*^
	FE × mean FS	—	—	—	−0.02	−2.67*⁣*^*∗∗*^

PhD students (*n* = 78)						
Mean DS	Gender	0.55	0.30	7.82*⁣*^*∗∗∗*^	−0.75	−0.44
	FE	—	—	—	−0.07	−0.72
	Mean FS	—	—	—	−1.19	−4.63*⁣*^*∗∗∗*^
	FE × mean FS	—	—	—	−0.05	−2.12*⁣*^*∗*^

Abbreviations: *β*, standardized coefficient; DS, depressive symptoms; FE, family environment; FS, family support.

*⁣* ^*∗∗∗*^ *p* < 0.001, *⁣*^*∗∗*^*p* < 0.01, *⁣*^*∗*^*p* < 0.05.

**Table 3:** Model fit, intercept, and slope of psychological resilience and depressive symptoms for master's students and PhD students.

Variable	CFI	RMSEA	SRMR	Means	
				Intercept	Slope
Master's students (*n* = 615)					
Psychological resilience	0.831	0.268	0.042	5.41*⁣*^*∗∗∗*^	0.32*⁣*^*∗∗∗*^
Depressive symptoms	0.935	0.152	0.040	5.28*⁣*^*∗∗∗*^	0.16*⁣*^*∗*^
PhD students (*n* = 78)					
Psychological resilience	0.905	0.213	0.031	6.15*⁣*^*∗∗∗*^	−0.07
Depressive symptoms	0.983	0.132	0.041	4.19*⁣*^*∗∗∗*^	0.12

*Note:* Standardized parameter estimates for intercept and slope were presented.

Abbreviations: CFI, comparative fit index; RMSEA, root mean square error of approximation; SRMR, standardized root mean square.

*⁣* ^*∗∗∗*^ *p* < 0.001, *⁣*^*∗*^*p* < 0.05.

**Table 4:** Direct effect coefficients for respective parallel process latent growth models.

Direct effects	Master's students (*n* = 615)			PhD students (*n* = 78)		
	*β*	*SE*	*p*	*β*	*SE*	*p*
FE→intercept of PR	0.57	0.04	＜0.001	0.46	0.17	＜0.01
FE→slope of PR	−0.24	0.06	＜0.001	0.11	0.20	0.583
FE→intercept of DS	0.10	0.06	0.079	0.01	0.56	0.980
FE→slope of DS	−0.24	0.10	＜0.05	0.15	1.52	0.920
Intercept of PR→intercept of DS	−0.88	0.04	＜0.001	−0.73	0.53	0.167
Intercept of PR→slope pf DS	0.25	0.10	＜0.05	0.29	1.43	0.067
Slope of PR→slope of DS	−0.85	0.15	＜0.001	−0.58	1.60	0.715

*Note:* Standardized parameter estimates were presented.

Abbreviations: DS, depressive symptoms; FE, family environment; PR, psychological resilience.

**Table 5:** Indirect effect coefficients for respective parallel process latent growth models.

Indirect effects	Master's students (*n* = 615)			PhD students (*n* = 78)		
	*β*	*p*	95%CI	*β*	*p*	95%CI
FE→intercept of PR→intercept of DS	−0.50	＜0.001	(−0.60, −0.42)	−0.34	0.525	(−6.92, −0.10)
FE→intercept of PR→slope of DS	0.14	＜0.05	(0.03, 0.27)	0.13	0.842	(−0.15, 4.91)
FE→slope of PR→slope of DS	0.21	＜0.01	(0.09, 0.37)	−0.06	0.893	(−0.55, 0.25)

*Note:* Standardized parameter estimates were presented.

Abbreviations: 95%CI, 95% confidence interval; DS, depressive symptoms; FE, family environment; PR, psychological resilience.

## Data Availability

The datasets presented in this article are not readily available because it used to support the findings of this study which are restricted by the the Ethics Committee, Department of Psychology, China University of Geosciences. In order to protect participant privacy, the data are prohibited from being made public. Requests to access the datasets should be directed to lilin@cug.edu.cn.
